# Correlation of serum leptin with levels of hemoglobin in hemodialysis

**Published:** 2012-07-01

**Authors:** Mahmoud Rafieian-Kopaei, Hamid Nasri

**Affiliations:** ^1^Medical Plants Research Center, Shahrekord University of Medical Sciences, Shahrekord, Iran; ^2^Section of Hemodialysis, Shahrekord University of Medical Sciences, Iran

**Keywords:** Leptin, Anemia, Hemodialysis, Hemoglobin

## Abstract

To examine the association of serum leptin level with anemia in hemodialysis, we investigated 36 patients (males: 21, diabetics: 11) under regular hemodialysis. For patients, complete blood counts, iron profile, serum leptin, and adequacy of hemodialysis were assessed. In this study a significant correlation of serum leptin with level of hemoglobin and body mass index was detected. An association between serum leptin and total iron binding capacity was observed. No correlation of serum ferritin with leptin level was seen. Our findings attest previous findings showing that greater serum leptin levels are associated with greater hemoglobin levels.

**Implication for health policy/practice/research/medical education:**

In a study on 36 hemodialysis patients, we observed a positive correlation between serum leptin level and body mass index and hemoglobin, which supports the theory of a reverse epidemiological role for leptin in regular hemodialysis patients.

## Introduction

Recent investigations have shown that leptin is cleared principally by the kidney. Therefore, serum leptin concentrations are increased in patients with chronic kidney disease and those undergoing maintenance dialysis (1-3). It has been thought that high serum leptin level may contribute to uremic anorexia and malnutrition (3-5). Serum leptin is generally elevated in chronic kidney disease and hemodialysis. Recent studies in dialysis patients suggest a paradoxically inverse relationship between elevated serum leptin and markers of nutritional status, a finding that is consistent with the theory of reverse epidemiology (2-8).

Usually chronic kidney disease patients develop anemia, which predominantly due to insufficient erythropoietin production by kidneys (9). Recently, studies have suggested a role for leptin in early stage of erythropoiesis and leptin has been found to stimulate hematopoietic stem cells in vitro (6-11).

## Objectives

Studies regarding the association of serum leptin with anemia in regular hemodialysis patients are quiet scarce. The aim of this study was to determine whether hyperleptinemia correlated with anemia markers in hemodialysis patients.

## Patients and Methods

This cross-sectional study was conducted on stable hemodialysis patients. We excluded patients who had active or chronic infection, and those using non-steroidal anti-inflammatory drugs (NSAID) or angiotensin converting enzyme (ACE) inhibitors.

### Laboratory assessments

Complete blood counts, serum iron, total iron binding capacity (TIBC), serum ferritin, serum creatinine, pre and post-dialysis blood urea nitrogen (BUN) was assessed. Blood samples were drawn after an overnight fast. Serum Leptin (normal range of values for males is 3.84 (1.79) and for females is 7.36 (3.73) ng/ml) was measured by enzyme-linked immunosorbent assay (ELISA) method using DRG kits- Germany.

To evaluate the efficacy of hemodialysis, the urea reduction rate (URR) was calculated from pre- and post-blood urea nitrogen (BUN) data (12). Body mass index (BMI) was calculated using the standard formula (post dialysis weight in kilograms/height in square meters; kg/m^2^) (13). Duration of hemodialysis treatment were obtained from the patients’ records. The duration of each hemodialysis session was 4 hours.

### Ethical approval

All patients signed the consent form for participation in this study. Research study was approved by the ethics committee of Shahrekord University of Medical Sciences, Iran.

### Statistical Analysis

Statistical analysis was performed on total hemodialysis (HD), females, males, diabetic and non-diabetic populations separately. Data were expressed as the mean ± standard deviation (SD). Student’s t-test was used to compare the study groups. Partial correlation and Pearson tests were used to evaluate statistical correlations. All statistical analyses were performed using SPSS (version 11.5). Statistical significance was determined at a p-value<0.05.

## Results

There was 36 hemodialysis patients (15 females, 21 males) consisting of 25 (11 females, 14 males) non-diabetic patients and 11 (4 females, 7 males) diabetic hemodialysis patients. Table 1, shows the patients’ data. The mean patients’ age was 47 (17) years. The median value of serum leptin in the study patients was 5.75 ng/ml; the median values of serum leptin in the diabetic and non-diabetic groups were 8.3 and 4 ng/ml, respectively. There was a significant positive correlation of serum leptin with hemoglobin levels (r= 0.36, p= 0.033). A significant positive correlation of serum leptin with BMI (r= 0.56, p< 0.001). There was no significant correlation between serum leptin and serum ferritin or total iron binding capacity in all patients or in the subgroups.

**Table 1:** Patients’ data

**Patients** **N=36**	**Minimum**	**Maximum**	**Mean±SD**	**Median**
**Age ( year)**	16	80	47±17	43
DH* ( month)	2	156	32±36	19
**Dialysis sessions**	36	1584	294±393	156
**URR ( %)**	39	76	59±9	57.5
**Leptin (ng/ml)**	0.10	73	9.4±14	5.75
**Hgb (g/dl)**	5	13	9±2	9
**HCT (%)**	14	40	28±6	2905
**Ferritin(ng/dl)**	35	1250	519±299	426
**Iron (micg/dl)**	10	1515	350±454	69
**TIBC (micg/dl)**	200	1875	968±562	1059
**BMI (kg/m** ^2^ **)**	16	34	21.8±4.5	21.5

*Duration of hemodialysis treatment

## Discussion

In this study, we found a significant positive correlation between serum leptin and hemoglobin and body mass index. Tungtrongchitr *et al*, considered, 214 overweight patients without kidney failure (body mass index ≥25). They found a significantly higher serum leptin level, mean corpuscular hemoglobin concentration and mean corpuscular volume in the overweights, in comparison to the control subjects (14). Previous studies in hemodialysis patients indicated that individuals with high serum leptin levels were more likely to lose weight (15-17). However, more recent studies of hemodialysis patients suggest a paradoxically inverse association between higher serum leptin and markers of nutritional status (6,7), a finding that was consistent with the theory of reverse epidemiology (8). Leptin is similar to serum albumin, which is a negative acute phase reactant in end-stage kidney failure patients (7).

A study conducted by Dedoussis *et al.* on 40 beta-thalassemia patients found that leptin might play some role in hematopoiesis (18). Kokot *et al.* assessed the influence of 12 months of Eprex therapy on plasma leptin in 15 hemodialysis patients and showed significantly lower leptin level after 3, 6, and 12 months of Eprex therapy as compared to the beginning of the study (19).

Of interest, hemodialysis patients with a high body mass index are less likely to experience severe anemia (20,21). Takeda *et al.* examined patients who could maintain high hemoglobin levels without the use of Eprex and found that in long-term hemodialysis patients, serum leptin levels correlated inversely with Eprex dose (22). Stenvinkel *et al.* also found that weekly Eprex doses correlated negatively with both body fat mass and serum leptin levels in patients with end-stage kidney failure not yet receiving dialysis (23). Hung *et al.* imagined that increasing caloric intake, body fat mass and leptin levels may enhance erythropoiesis in long-term hemodialysis patients. Hyperleptinemia may reflect better nutritional status and Eprex response in hemodialysis patients. Increasing energy intake corrects erythropoiesis, which may be partly mediated by an increase in serum leptin levels (24). Increased leptin levels could both improve the erythropoietic response and reduce Eprex dose when greater body fat mass is achieved by caloric supplementation (24). While, leptin is considered an “appetite inhibitor” in the general population, its role in end-stage kidney failure patients is somewhat unconventional. Serum leptin is generally elevated in end-stage kidney failure patients, but this has not been observed in anorexia. Leptin has been shown to act synergistically with erythropoietin to stimulate end-stage colony-forming-unit erythroid in humans (25).

## Conclusion

In this study, we detected a positive correlation between serum leptin level with body mass index and hemoglobin level, which supports the theory of a reverse epidemiological role for leptin in the regular hemodialysis patients.

## Authors’ contributions

MRK and HN wrote the manuscript equally.

## Conflict of interests

The authors declared no competing interests.

## Ethical considerations

Ethical issues (including plagiarism, misconduct, data fabrication, falsification, double publication or submission, redundancy) have been completely observed by the authors.

## Funding/Support

This study was supported by a grant from Shahrekord University of Medical Sciences.
